# P-1374. Syphilis in an American Indian Population: A Descriptive Analysis of Cases

**DOI:** 10.1093/ofid/ofae631.1550

**Published:** 2025-01-29

**Authors:** Andrea L Blair, Ashley L Comiford, Jorge Mera

**Affiliations:** Cherokee Nation, Tahlequah, Oklahoma; Cherokee Nation, Tahlequah, Oklahoma; Cherokee Nation, Tahlequah, Oklahoma

## Abstract

**Background:**

Syphilis, a sexually transmitted infection, caused by the bacterium *Treponema subspecies pallidum*. Because of its numerous varied clinical presentations, it has earned the term "the great imitator and mimicker." Between 2017 and 2021, syphilis rates in the US increased by 72%, and Oklahoma cases increased by 223%. Further, American Indian/Alaska Natives (AI/AN) are disproportionately burdened by the increase in syphilis rates.

The objective of this presentation is to describe demographic characteristics of AI individuals diagnosed with syphilis between 2016 and 2023 within the Cherokee Nation Health Services (CNHS) system.

**Methods:**

We extracted patient data collected through the CNHS electronic health record system. Individuals were included in this analysis if they had a positive syphilis result from January 1, 2016 through July 1, 2023. We analyzed the data using descriptive statistics including counts and percentages.

**Results:**

Over the study period, 375 individuals were diagnosed with syphilis. The majority of individuals were 34 years or younger (61.5%), 50% of were female, 69.3% had a non-emergency or urgent care healthcare visit within the previous 12 months. About 45% had insurance, and 84% were single. Nearly 85% of individuals resided in a county with a Cherokee Nation clinic and 53.3% received treatment documented in the EHR. Most individuals were diagnosed during the COVID-19 pandemic (75.2%) and 45.1% were diagnosed through the emergency or urgent care departments. About 13% had a diagnosis of HCV, 30% had more than one other STI, and 16% of individuals received HIV PrEP treatment.

**Conclusion:**

In the Cherokee Nation, syphilis primarily affects women of childbearing age, with a significant portion diagnosed in urgent care and emergency department settings, yet only 53% receive treatment. Investigating the reasons behind such low treatment rates and devising interventions to enhance them will be pivotal in curbing the surge in syphilis cases. Crucially, identifying demographic and other pertinent factors that could pinpoint those vulnerable to syphilis is imperative. Further research should concentrate on delineating risk factors for syphilis in this demographic and implementing preventive measures accordingly.

**Disclosures:**

**All Authors**: No reported disclosures

